# IL-10 family cytokines in chronic rhinosinusitis with nasal polyps: From experiments to the clinic

**DOI:** 10.3389/fimmu.2022.947983

**Published:** 2022-08-08

**Authors:** Lijia Xuan, Nan Zhang, Xiangdong Wang, Luo Zhang, Claus Bachert

**Affiliations:** ^1^ Department of Otolaryngology Head and Neck Surgery, Beijing TongRen Hospital, Capital Medical University, Beijing, China; ^2^ Beijing Key Laboratory of Nasal Diseases, Beijing Institute of Otolaryngology, Beijing, China; ^3^ Upper Airways Research Laboratory, Ghent University, Ghent, Belgium; ^4^ Department of Allergy, Beijing TongRen Hospital, Capital Medical University, Beijing, China

**Keywords:** IL-10, CRSwNP, epithelium, infection, therapy

## Abstract

Chronic rhinosinusitis with nasal polyps (CRSwNP) is considered a nasal sinus inflammatory disease that can be dominated by immune cells and cytokines. IL-10 family cytokines exert essential functions in immune responses during infection and inflammation. Recently, the understanding of the roles of the IL-10 family in CRSwNP is being reconsidered. IL-10 family members are now considered complex cytokines that are capable of affecting epithelial function and involved in allergies and infections. Furthermore, the IL-10 family responds to glucocorticoid treatment, and there have been clinical trials of therapies manipulating these cytokines to remedy airway inflammatory diseases. Here, we summarize the recent progress in the understanding of IL-10 family cytokines in CRSwNP and suggest more specific strategies to exploit these cytokines for the effective treatment of CRSwNP.

## Introduction

Chronic rhinosinusitis (CRS) is a persistent inflammatory disease of the nasal and paranasal sinuses lasting for more than 12 weeks, with patients presenting two or more symptoms, including nasal congestion, loss of smell, nasal discharge and facial pain or pressure (1). CRS is generally divided into two phenotypes: CRS with nasal polyps (CRSwNP) and CRS without nasal polyps (CRSsNP) based on whether nasal polyps are found during endoscopy or during surgery (1). Endotyping provides a more comprehensive approach than phenotyping by emphasizing pathophysiological factors involved in CRS (1). Most patients with CRSsNP present a type 1 immune response that dominated by T helper (Th) 1 cell accompanied by tissue fibrosis with an increase in TGF-β level (2). The inflammatory status in CRSwNP varies with the location and environment. In Europe and North America, most inflammatory responses involve eosinophils, while few eosinophils and more neutrophils are observed in the inflammatory responses in Asian patients (3). Based on the types of cytokines secreted from Th cells, including interleukin (IL)-1, IL-4, IL-5, IL-17 and IFN-γ, CRSwNP is commonly divided into Th1, Th2 and Th17 endotypes (4). Approximately 80% of nasal polyps in patients from the Western countries show type 2 characteristics compared with approximately 20 to 60% of nasal polyps in patients from China, South Korea and Thailand (3). Western patients with CRSwNP have significantly increased levels of Th2 cytokines and transcription factors, but patients with CRSwNP in China show a predominant Th1/Th17 pattern (5). Differences in inflammatory patterns of sinonasal inflammation may be impacted by genetic or environmental factors such as pollution; however, the local mucosal colonization likely is the most important factor to determine differences in inflammation (5, 6).

IL-10 was first identified in 1989, and was initially described as a Th2 cell cytokine (7). A group of cytokines with similar protein structures and receptor complexes to IL-10 are also known as IL-10 family cytokines, including IL-19, IL-20, IL-22, IL-24, IL-26, IL-28A, IL-28B, and IL-29 (8). Despite the similarities in structures, members of the IL-10 family possess different biological functions in the immune system. They are secreted by innate and adaptive immune cells including monocytes, B cells, T cells, NK cells or macrophages, as well as structural cells, such as epithelial and endothelial cells (7). IL-10 family cytokines have versatile immune-mediating functions, and many attempts have been made to use IL-10 family members in therapeutic strategies for autoimmune diseases, cancers and inflammatory diseases (9).

Based on accumulating evidence, the heterogeneity in CRS manifestations may be explained by a variety of disparate molecular and cellular pathways that result in mucosal inflammation of CRS. The improved understandings of different pathophysiological mechanisms of CRS facilitated the identification of disease variants as endotypes (10). However, no consistent effect of IL-10 family cytokines in CRSwNP has been reported. Therefore, the current review will focus on the most common and important roles of IL-10 family cytokines in CRSwNP.

## IL-10 family cytokines, receptors and signaling pathways

**Figure 1:**
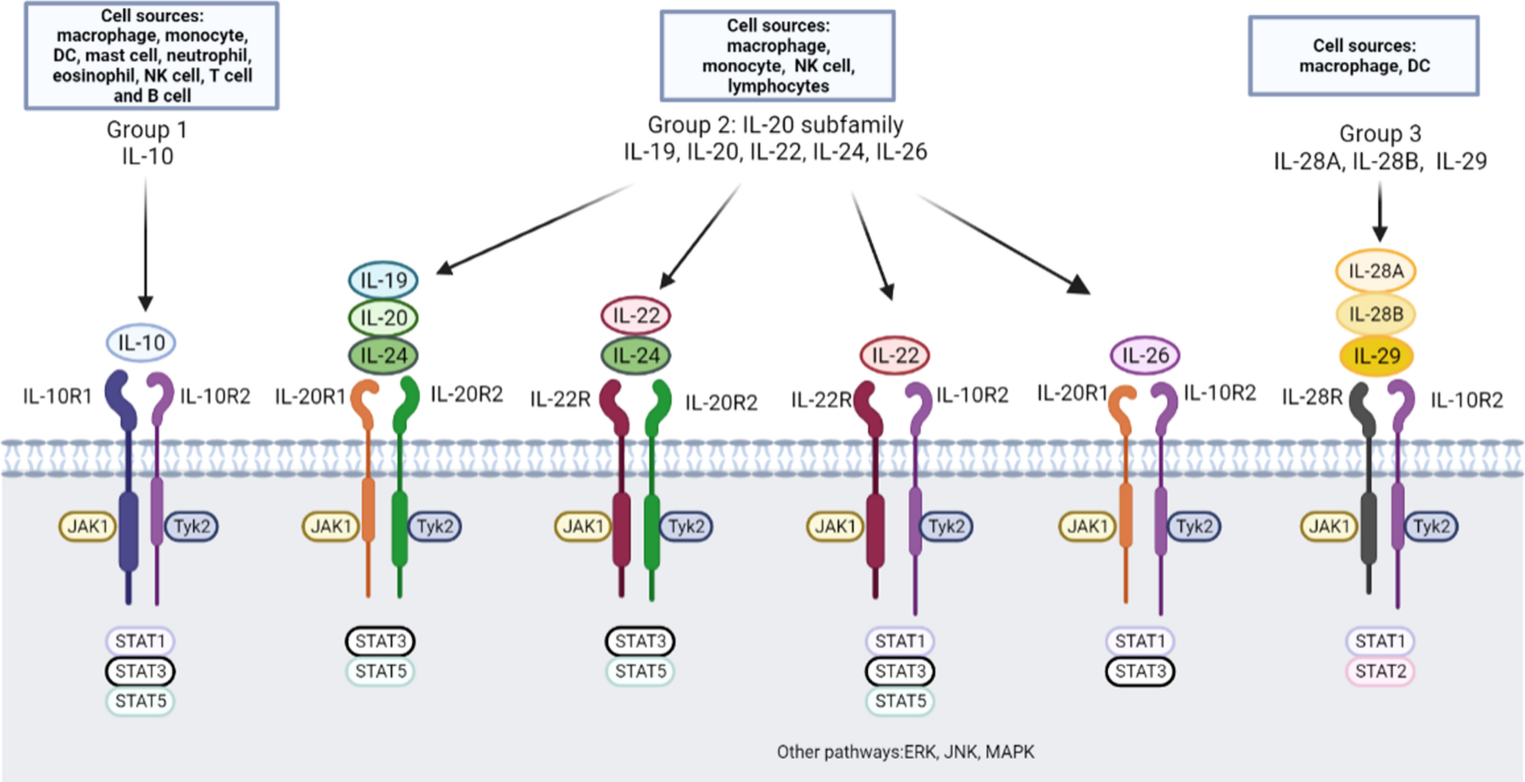
IL-10 family cytokines, receptors and downstream pathway. Nine cytokines in the IL-10 family have been identified, and these cytokines can be divided into three groups: the IL-10, IL-20 subfamily and group 3. DC, dendritic cells; NK cell, natural killer cell; JAK, Janus tyrosine kinase; Tyk, tyrosine kinase; STAT, signal transducer and activator of transcription; ERK, extracellular signal-regulated kinase; JNK, c-Jun N-terminal kinase; MAPK, mitogen-activated protein kinase.

IL-10 family cytokines consist of IL-10, IL-19, IL-20, IL-22, IL-24, IL-26, IL-28A, IL-28B, and IL-29 (11), which are categorized into three subgroups based on similarities concerning the structures and genomic location of protein-coding genes, primary and secondary protein structures, and receptor complex utilization (7) (**Figure 1**). The first group includes only IL-10, which is an anti-inflammatory cytokine (12) and binds to the IL-10 receptor 1 (IL-10R1) and IL-10R2 subunits. The second group, also called the IL-20 subfamily, contains IL-19, IL-20, IL-22, IL-24 and IL-26. IL-19, IL-20, and IL-24 bind to a cell surface receptor complex composed of two chains, IL-20R1 and IL-20R2 (13). IL-20R2 also pairs with IL-22R to form a receptor complex that binds IL-20 and IL-24 but not IL-19 (13). In addition, IL-22 binds to a heterodimeric receptor complex composed of IL-22R and IL-10R2 (14). IL-26 binds to the IL-20RA/IL-10RB two-chain receptor complex (15). The third group is composed of IL-28A, IL-28B, and IL-29, which are related to type III interferons (IFN-λs) (16). IL-28A (IFN-λ2), IL-28B (IFN-λ3), and IL-29 (IFN-λ1) share the same IL-10R2 chain with IL-10 and employ IL-28R as the α chain to induce similar downstream biological effects as type I IFNs (17). The ligand-binding chains for IL-22, IL-26, IL-28A/B, and IL-29 share a common β chain, IL-10R2, which they employ to assemble their active receptor complexes, similar to IL-10 (11, 18).

Downstream signaling pathways of IL-10 family cytokines have been extensively studied (7, 11). The best-characterized signaling pathway of IL-10 is the JAK/STAT axis (19). The downstream interaction of IL-10 with its receptors engages Janus tyrosine kinase 1 (JAK1) and tyrosine kinase 2 (Tyk2), which are associated with IL-10R1 and IL-10R2, respectively (20, 21). JAK1 and Tyk2 induce tyrosine phosphorylation and activation of the latent transcription factors signal transducer and activator of transcription 1 (STAT1), STAT3, and sometimes STAT5 (22). IL-22 is currently the most well studied IL-20 subfamily member and induces activation of the JAK/STAT pathway, phosphorylation of Jak1 and Tyk2 (23), and STAT1, STAT3, and STAT5 activation. IL-22 also activates the extracellular signal-regulated kinase (ERK), c-Jun N-terminal kinase (JNK), and p38 mitogen-activated protein kinase (MAPK) pathways in a rat hepatoma cell line (23) and a human intestinal epithelial cell line (24).

Despite similarities in specific features, IL-10 family members exert different biological effects. IL-10 was first described as a Th2 cytokine (25), but further evidence suggested that IL-10 may be produced by most immune cell types, including innate (including macrophages, monocytes, dendritic cells (DCs), mast cells, neutrophils, eosinophils and natural killer (NK) cells) and adaptive (including CD4+ T cells, CD8+ T cells and B cells) immune cells (26). IL-20 subfamily cytokines are mainly produced by immune cells, such as monocytes, lymphocytes, NK cells, macrophages and fibroblasts (27). In contrast to IL-10 family cytokines, IL-20 subfamily cytokines primarily function in epithelial cells, protecting them from extracellular pathogen invasion, and enhancing the wound-healing activities (28). The cellular sources of the third group cytokines can be produced by leukocytes (29). IL-28R generation is restricted to cells of epithelial origin, including hepatocytes and myeloid lineage cells, such as DCs and macrophages (30). IFN-λs primarily act on epithelial cells by binding to IL-28R/IL-10R2 to activate JAK/STAT signaling and have exert antiviral effects similar to those of type I IFNs (31, 32). Despite the tissue-protective function of the IL-10 family, uncontrolled tissue repair processes may cause the disorders in inflammatory diseases.

## Differential expression of IL-10 family cytokines in individuals with CRSwNP

Immunologic heterogeneity in different regions among patients with the same disease has been observed in individuals CRSwNP (3). Different immunoregulatory activities have the capacity to participate in CRSwNP mechanisms. Heterogeneity is also observed in the expression of the IL-10 family. No significant difference was observed for IL-10 between the tissue homogenates of the nasal polyps and turbinate tissue from the control subjects in a study conducted in Germany (33). However, in a study conducted in Korea, the serum IL-10 level in CRSwNP patients was significantly lower than that in healthy people, but no difference was observed between eosinophilic CRSwNP and those with non-eosinophilic CRSwNP (34). In new research from Brazil, the level of IL-10 protein in nasal polyps was found to be significantly lower than that in mucosa from the control group (35). IL-10 level was inversely correlated with olfactory function in patients with CRS (36). The IL-19 mRNA was significantly overexpressed in the nasal polyp group compared with that in both the CRSsNP and control groups (37). IFN-λs are present at lower levels in the nasal mucosa of CRS patients than in healthy controls (38). The IL-22 expression level of was not different between control and CRSwNP groups, but it was significantly increased in CRSsNP group compared to the control and CRSwNP groups (39). According to a factor analysis based on principal component analysis, IL-22 level positively correlated with type 2 cytokine levels and the Lund-Mackay CT score, which is a degree of sinus opacification ranges from 0 to 24 (39). Whereas another study compared the levels of cytokines across three different age groups and found significantly higher level of IL-22 in the mucosa from CRSwNP than in the control group across all age groups and correlated with the severity of clinical symptoms (40).

## IL-10 family cytokines play roles in the mechanisms of CRSwNP

### The effects of IL-10 family cytokines on epithelial function

**Figure 2:**
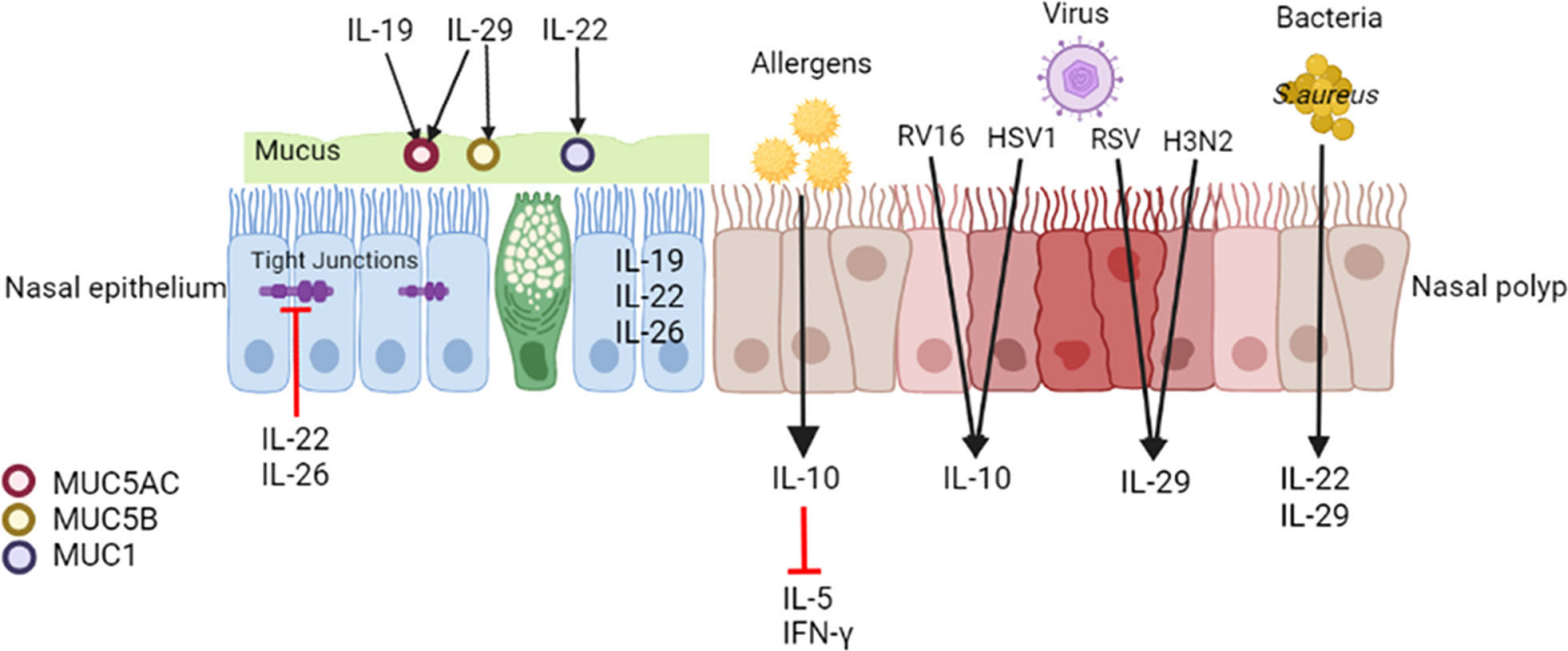
Effects of IL-10 family cytokines on CRSwNP. IL-19, IL-22 and IL-26 are expressed in the nasal epithelium. IL-22 and IL-26 disrupt the epithelial barrier of nasal epithelial cells from patients with CRS. IL-19 upregulates MUC5AC expression in the epithelium in CRS, and IL-29 upregulates MUC5AC and MUC5B synthesis in the healthy nasal mucosa. IL-22 enhances MUC1 in nasal polyp cells. Upon encountering with inhaled allergens, epithelial cells may release IL-10 and consecutively decrease allergen-induced IL-5 and IFN-γ production in nasal polyps. Viral and bacterial infections modulate the expression of IL-10 family members. Both RV16 and HSV1 elevate IL-10 expression in nasal polyps. Both RSV and H3N2 increase IL-29 expression in the nasal epithelium of CRS. SEB induces IL-22 production, and *S. aureus* infection increases IL-29 expression in nasal polyps. MUC, mucin; NP, nasal polyp; SEB, staphylococcal enterotoxin B.

The mucosal epithelium maintains tissue defense functions through mucociliary action, mucus production, and ion transport, and it serves as the first barrier for pathogen defense (41). Intercellular epithelial junctions, including tight junctions, adherent junctions, and desmosomes, determine epithelial barrier integrity (42). Damaged integrity of the nasal mucosal epithelium is a common feature of CRS, which features increased epithelial permeability and reduced antimicrobial substances and responses (43) (**Figure 2**).

Some studies have demonstrated that IL-20 subfamily cytokines affect the nasal epithelium. Pace et al. (37) observed increased expression of the IL-19 mRNA and protein in the epithelium of individuals with nasal polyps, which may alter the squamous meta plasticity of the nasal epithelium of patients with CRSwNP mediated by tyrosine phosphorylation events. IL-22 and IL-22R1 are mainly expressed in infiltrating inflammatory cells and epithelial cells, respectively, in nasal polyps (44). In an air-liquid interface model of nasal epithelial cells from patients with CRS, IL-22 and IL-26 disrupted the epithelial barrier, as evidenced by a loss of transepithelial electrical resistance (TEER), increased paracellular permeability, and disruption of the tight junction protein zona occludens-1 (ZO-1) (45).

Mucus is a barrier lining the respiratory epithelium consisting of water, proteins and macromolecules that package and remove inhaled foreign substances such as microbes and pollutant particles (46). The overproduction of purulent mucus is typically related to symptoms of CRS, and the secretions are more viscous, elastic, and adhesive than normal nasal mucus (47). A quantitative analysis of cytokines in mucus may represent a novel method to link symptoms with an objective measure of inflammation (48). The level of IL-10 in mucus collected from the olfactory cleft and middle meatus of patients was not different among CRSwNP, CRSsNP and control groups, but it correlated with reduced olfactory identification scores (49) and olfactory clef opacification on CT (50). Mucin (MUC) glycoproteins comprise the major part of mucus, and they are critical for the local defense of the airway (46). MUC5AC and MUC5B presented at significantly higher levels in the IL-5(+) CRSwNP group than in the IL-5(-) CRSwNP and healthy nasal mucosa groups (51). Pretreatment with rhIL-19 upregulates MUC5AC expression in PHNECs derived from patients with CRS, which may occur *via* the STAT3 pathway (52). IL-29 increases MUC5AC and MUC5B synthesis in healthy nasal mucosa to enhance the antibacterial function of the epithelium (53). MUC1 expression is downregulated in patients with CRSwNP who are resistant to oral glucocorticoids (GCs), and MUC1 participates in mediating corticosteroid receptor α nuclear translocation (54). IL-22 may significantly increase MUC1 mRNA expression in nasal polyp dispersive cells (44). Therefore, IL-22 may reverse the corticosteroid resistance of some difficult-to-treat nasal polyps.

### The IL-10 family and allergies

A high prevalence of atopy has also been observed in patients with CRSwNP, and it is potentially associated with disease severity (55). IL-10 directly interacts with and downregulates IL-2 and IFN-γ production by Th1 cells and IL-4 and IL-5 production by Th2 cells (56). Nasal polyp tissues harvested from CRSwNP patients sensitized to aeroallergens were digested to single cell suspensions and stimulated with varying concentrations of cat, grass or house dust mite allergen to which the patients were sensitized, significantly increases IL-10 production by cell suspensions, and neutralization of IL-10 significantly increases allergen-specific IL-5 and IFN-γ production by nasal polyp cells (57) (**Figure 2**).

### The IL-10 family and infections

Viral infections may contribute to and exacerbate CRS (**Figure 2**). Virus-sensing molecules induce the production and secretion of nuclear factor-κB (NF-κB), IFN-β, TNF-α, IL-1β, IL-6, and IL-8, which have the potential to recruit neutrophils and macrophages (58). Based on functional diversity in phenotype, macrophages could be classified into classically-activated pro-inflammatory M1 and alternatively-activated anti-inflammatory M2, which are additionally sub-categorized into M2a, M2b, M2c and M2d (59). IL-4 and IL-13 may induce the polarization of M2 macrophages, and the secretion of cytokines such as IL-10 may also activate this process (60). M2a, M2b and M2c are related to IL-10. The research found the combination of IL-10 and IL-4 enhanced the expression of the M2a-related genes more than either IL-4 or IL-10 alone in mice bone marrow-derived macrophages (BMDMs), and increased eosinophil migration (61). M2b cells are the main cellular source for IL-10 playing an important role in inflammation resolution (62). M2c could be activated by IL-10 and TGF-β, and IL-10-stimulated M2c has a higher phagocytic capacity for apoptotic cells *in vitro* (63). The increased IL-10 content induced by these macrophages suggests their anti-inflammatory properties (64).

The most common viruses that infect the upper airway and cause CRS are similar to those causing acute rhinosinusitis (ARS), including human rhinovirus (HRV), respiratory syncytial virus (RSV) and influenza virus (58). When the uncinate mucosa of patients with allergic CRS was treated with RV16, a significantly elevated IL-10 level was detected (65). RSV is a major pathogen that primarily infects the airway epithelium in a healthy nasal turbinate and nasal polyp explant *ex vivo* model. After 48 and 72 h of infection with herpes simplex virus 1 (HSV1), a type of RSV, a significantly higher level of released IL-10, rather than IFN-λ, was detected in the supernatant of nasal polyps compared to control mucosa (66). In addition, the air-liquid epithelium model of well-differentiated primary pediatric nasal (WD-PNECs) generated from nasal brushes and bronchial epithelial cells (WD-PBECs) generated from bronchial brushes induced more IL-29 generation in the culture medium after infected with RSV (67). Luukkainen et al. (68) assessed an *in vitro* co-culture model in transwells of influenza A virus H3N2 infection of nasal epithelium isolated from biopsies of patients with CRSwNP and observed a significant increase in IL-29 in the epithelium after infection.

In addition to viruses, bacteria affect CRS, and an imbalanced microbiota might be one of the initial causes of the chronic immune response and inflammation (69). The abundance of *Staphylococcus aureus* (*S. aureus*) is increased in individuals with CRS (70), and *S. aureus* is more frequently detected in the nasal mucosa of CRSwNP than in that of patients with CRSsNP (71). Staphylococcal enterotoxin B (SEB) is a specific *S. aureus* IgE superantigen that may skew the immune response toward a Th2 response and trigger nasal polyps formation (72). IL-10 family cytokines may function against bacteria. After exposure to SEB, the IL-10 level was not significantly different between the NP or uncinate tissue, but it significantly negatively correlated with the eosinophil count (73). And the presence of IL-10 may neutralize the SEB-induced IL-13 and IFN-gamma production by NP cells (73). Toll-like receptor (TLR)-mediated signals are known to be associated with the pathogenesis of CRSwNP (74). Polycytidylic acid (poly (IC)) is the ligand for TLR3, and exposure to poly (IC) selectively induces IL-10, but not IL-5, IL-13, IFN-γ or IL-17A, production by nasal polyp cells. Neutralization of IL-10 significantly increased the production of SEB-induced IL-5, IL-13, IFN-γ and IL-17A by nasal polyp cells exposed to poly (IC), which illustrates that TLR3 signaling regulates SEB-induced cytokine production in CRSwNP *via* IL-10 (75). SEB-induced IL-22 production from nasal polyp cells significantly and negatively correlated with the degree of local eosinophilia and the postoperative CT score, and it positively correlated with the forced expiratory volume in 1 s (FEV1)/forced vital capacity (FVC) ratio (44). *S. aureus* infection increased IL-29 expression in nasal polyps (53). In healthy nasal mucosa, IL-29 favors *S. aureus* clearance by enhancing the antibacterial function of macrophages through the modulation of the ROS/JAK/STAT signaling pathway, but this phenomenon does not occur in individuals with CRSwNP (53). This notion is consistent with the reduced capacity to phagocytose *S. aureus* and reduced antibacterial function of macrophages in CRSwNP (76).

## The IL-10 family and treatment of CRSwNP

### The effect of GC treatment on the IL-10 family

GCs are a common pharmacotherapy with anti-inflammatory effects used to treat CRSwNP (77). GCs are known to reduce the numbers of eosinophils, mast cells, T lymphocytes, and DCs in the airway (78). Administration of GCs increases the number of CD4+ FOXP3+ Tregs in CRSwNP (79, 80). Kou *et al.* (79) found that TGF-β1, IL-10, p-Smad2, SOCS3, and FOXP3 levels were increased in nasal polyps compared to healthy nasal mucosa after GC treatment. Li et al. (80) also reported that after intranasal GC treatment of CRSwNP patients, Foxp3 and IL-10 expression were increased significantly in nasal polyp specimens after intranasal GC treatment. Similar results were reported in ECRSwNP: TGF-β and IL-10 expression increased after transnasal budesonide nebulization (81). All these results suggest that both IL-10 and Tregs are involved in suppressing inflammation in individuals with CRSwNP after GC treatment.

### The IL-10 family as therapeutic agents in airway inflammatory diseases

**Figure 3:**
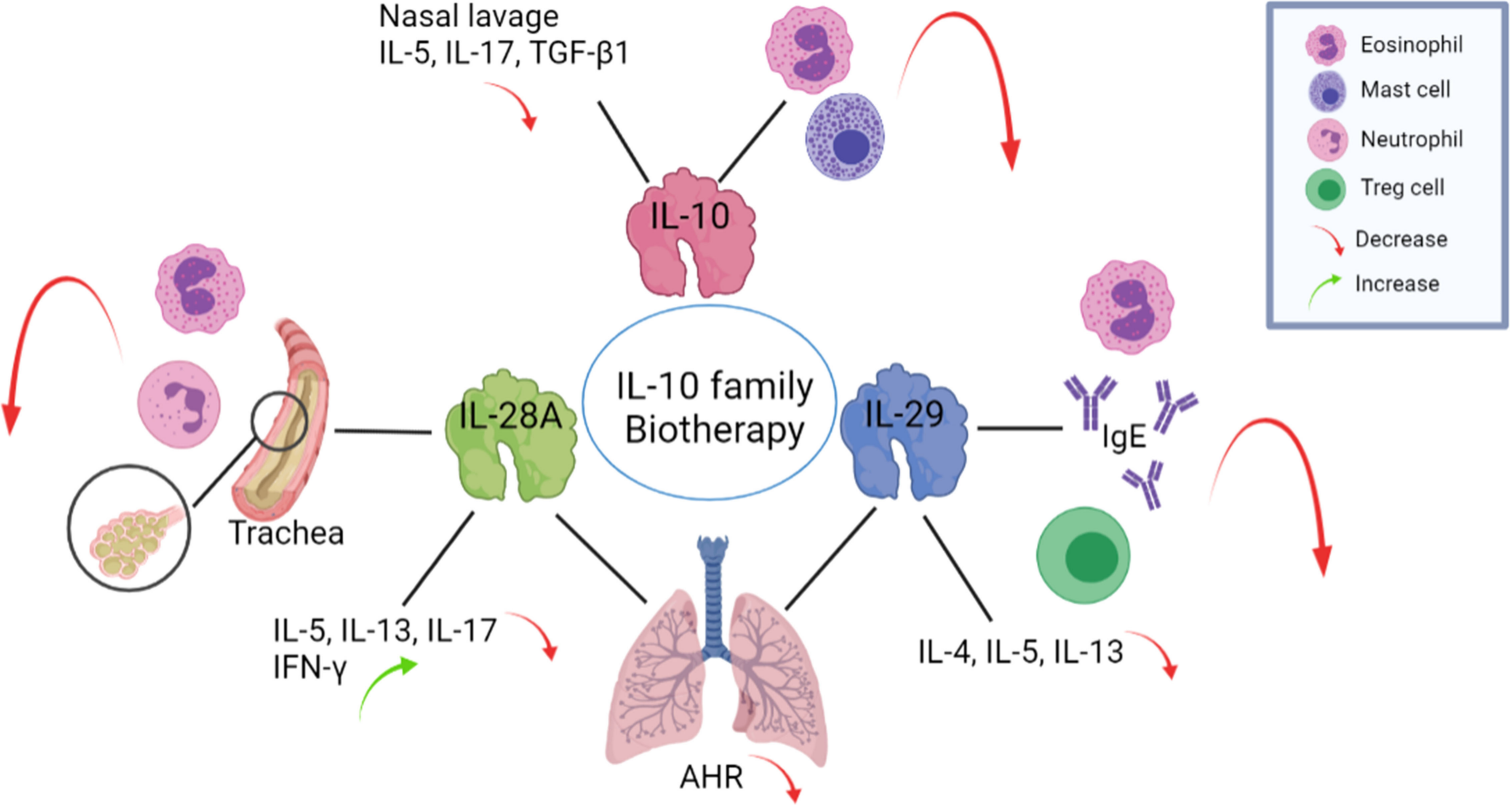
The application of IL-10 family members may exert beneficial effects on airway inflammatory diseases. Exogenous IL-10 administration reduces the infiltration of eosinophils and mast cells in the nasal mucosa and reduces IL-5, IL-17, and TGF-β1 levels in nasal lavage fluid. Intranasal administration of IL-28A to OVA-challenged mice suppresses the number of eosinophils and neutrophils in the BALF and reduces IL-5, IL-13 and IL-17 levels but increases IFN-γ level in lung-draining mediastinal lymph nodes. The administration of IL-29 to an OVA mouse model decreased eosinophil count, serum IgE level, induction of Tregs production and the expression levels of the Th2 cytokines IL-4, IL-5, and IL-13. Both IL-28A and IL-29 reduce AHR levels. BALF: bronchoalveolar lavage fluid, AHR: airway hyperresponsiveness, OVA: ovalbumin.

Although many treatments have been applied to airway diseases, many patients are not cured using traditional approaches. For CRS, patients who do not reach an acceptable level of control despite appropriate surgery and intranasal or systemic GC treatment in the last year can be considered to have difficult-to-treat CRS (82). Similarly, mild asthma is controlled by inhaled GCs, but adequate control of moderate to severe asthma is not easily achieved using these therapies, and thus new and better treatments are needed (83). Due to the anti-inflammatory function of IL-10 family cytokines, they may serve as therapies for airway inflammatory diseases (**Figure 3**).

In ovalbumin (OVA) challenged Allergic Rhinitis (AR) murine model, 7 days of continuous exogenous administration recombinant mouse (rm) IL-10 reduced the infiltration of eosinophils, mast cells and local IL-10 positive cells in the nasal mucosa, and increased total IL-10 levels but markedly reduced IL-5, IL-17, and TGF-β1 levels in nasal lavage fluid (84). After a restrictive IL-10 virus vector was inserted into an allergen-induced nasal allergic mouse model, the inflammatory response, including edema and infiltration of mononuclear cells, neutrophils and eosinophils, as well as increases in cytokine concentrations in nasal secretions, were significantly decreased (85). Treating blood monocyte-derived DCs with IL-29 led to the induction of IL-2-dependent proliferation of Tregs (86), which may reverse Th cell- mediated inflammation.

IFN-λs serve as therapeutic agents to help treat asthma because of the induction of Th1 cytokines productions, thereby antagonizing the effects of Th2 cytokines and activating functions similar to those of IFN-γ (87). Intranasal administration of recombinant IL-28A to mice during OVA challenge effectively decreases the number of eosinophils and neutrophils in the bronchoalveolar lavage fluid (BALF) and inhibits the infiltration of leukocytes in the lung and mucus secretion from goblet cells (30). IL-28A treatment significantly reduces IL-5, IL-13 and IL-17 levels in lung-draining mediastinal lymph nodes, upregulates IFN-γ, and ameliorates lung function by reducing airway hyperresponsiveness (AHR) (30). These data support a potent therapeutic effect of IL-28A treatment on allergic airway disease. Li et al. (88) administered an intranasal adenovirus expressing IL-29 to a murine model of OVA-induced asthma and found that IL-29 significantly decreased the severity of AHR and attenuated allergic airway inflammation by decreasing eosinophil counts, serum IgE levels, the activation of Tregs and expression of the Th2 cytokines IL-4, IL-5, and IL-13. After the administration of IL-29, the production of Treg is significantly induced in the mouse spleen to participate in suppressing inflammatory (88). Although the OVA-induced murine model is the classical model of asthma, it reflects mainly airway inflammation instead of most of the features of the disease due to associated immune tolerance (89). Using the OVA model cannot mimic all the symptoms of chronic asthma, but from the research above, the IL-10 family indeed could affect airway inflammation. In summary, these findings related to airway inflammation support the hypothesis that strategies targeting IL-10 family members might be beneficial for the development of future therapies.

IL-10 family cytokines have been utilized in clinical trials in many diseases such as Crohn’s disease, rheumatoid arthritis, acute pancreatitis, psoriasis, systemic lupus and pancreatic cancer (90). Yet no therapy or clinical trial of the IL-10 family in human airway inflammation diseases has been approved or proceeded to date, all the findings were in animal models or *in vitro*. Indeed, no animal model is available that mimics all the symptoms of diseases in human, so the biotherapy functions of the IL-10 family in human airway inflammation diseases still demand prompt study.

## Conclusions

IL-10 family cytokines possess diverse functions in CRSwNP that are related to the epithelium, the effect of allergens, and viral or bacterial infections, and respond to GCs. IL-10 family cytokines have been applied in few clinical trials of airway inflammation therapies. Therefore, IL-10 family cytokines may provide therapeutic benefits in CRSwNP.

## Author contributions

Conception and design: LZ and CB Manuscript writing: LX, NZ, and XW Manuscript revising: LX, NZ, LZ and CB. Final approval of manuscript: all authors.

## Funding

This work was supported by grants from the national natural science foundation of China (82101189, 82171110), the program for Changjiang scholars and innovative research team (IRT13082), CAMS Innovation Fund for Medical Sciences (2019-I2M-5-022), and Beijing municipal science and technology project (Z181100001618002).

## Conflict of interest

The authors declare that the research was conducted in the absence of any commercial or financial relationships that could be construed as a potential conflict of interest.

## Publisher’s note

All claims expressed in this article are solely those of the authors and do not necessarily represent those of their affiliated organizations, or those of the publisher, the editors and the reviewers. Any product that may be evaluated in this article, or claim that may be made by its manufacturer, is not guaranteed or endorsed by the publisher.
